# Fault Detection and Classification of CIGS Thin-Film PV Modules Using an Adaptive Neuro-Fuzzy Inference Scheme

**DOI:** 10.3390/s23031280

**Published:** 2023-01-22

**Authors:** Reham A. Eltuhamy, Mohamed Rady, Eydhah Almatrafi, Haitham A. Mahmoud, Khaled H. Ibrahim

**Affiliations:** 1 Mechanical Engineering Department, Faculty of Engineering, Helwan University, Cairo 11795, Egypt; 2 Mechanical Engineering Department, Ahram Canadian University, Cairo 12451, Egypt; 3 Mechanical Engineering Department, Faculty of Engineering at Rabigh, King Abdulaziz University, Jeddah 21589, Saudi Arabia; 4 Industrial Engineering Department, College of Engineering, King Saud University, Riyadh 11421, Saudi Arabia; 5 Electrical Power Department, Faculty of Engineering, Fayoum University, El-Fayoum 63514, Egypt

**Keywords:** CIGS thin film, PV modules, adaptive neuro-fuzzy inference system, operating power ratio

## Abstract

The use of artificial intelligence to automate PV module fault detection, diagnosis, and classification processes has gained interest for PV solar plants maintenance planning and reduction in expensive inspection and shutdown periods. The present article reports on the development of an adaptive neuro-fuzzy inference system (ANFIS) for PV fault classification based on statistical and mathematical features extracted from outdoor infrared thermography (IRT) and I-V measurements of thin-film PV modules. The selection of the membership function is shown to be essential to obtain a high classifier performance. Principal components analysis (PCA) is used to reduce the dimensions to speed up the classification process. For each type of fault, effective features that are highly correlated to the PV module’s operating power ratio are identified. Evaluation of the proposed methodology, based on datasets gathered from a typical PV plant, reveals that features extraction methods based on mathematical parameters and I-V measurements provide a 100% classification accuracy. On the other hand, features extraction based on statistical factors provides 83.33% accuracy. A novel technique is proposed for developing a correlation matrix between the PV operating power ratio and the effective features extracted online from infrared thermal images. This eliminates the need for offline I-V measurements to estimate the operating power ratio of PV modules.

## 1. Introduction

A crucial technology for a sustainable energy supply is the adoption of PV modules. According to recent statistics, the reliance on PV modules’ capacity has increased globally from 17 GW in 2010 to 139 GW in 2020 and has reached 760 GW at the end of 2020 [1]. Several techniques have been proposed for fault detection and diagnosis in PV modules; examples of these techniques include visual examination, infrared thermography (IRT), electroluminescence (EL), photoluminescence (PL), and measurements of static characteristics [2]. Based on the previously mentioned PV fault detection techniques, different types of PV module defects have been addressed, such as cracks, delamination, burn marks, potential-induced degradation (PID), soiling, snail trails, hotspots, faulty interconnections, back sheet defects, corrosion, and shunts [3,4,5]. The continuous, safe, reliable, and effective functioning of PV systems depends on the development of automatic diagnosis and classification techniques for PV monitoring systems. The huge number of solar modules used in large-scale PV facilities has made it difficult to detect and classify faults [6,7,8,9]. The automation of detection, diagnoses, and classification of PV system faults, using artificial intelligence (AI) approaches, has attracted a great deal of research interest. Many AI techniques have been suggested in the literature, such as particle swarm optimization [10], ant colony optimization [11], support vector machine [12], k-nearest neighbors [13], linear regression [14], decision tree [15], naive Bayes (NB) [16], neural network systems [17], and fuzzy logic [18]. Additionally, hybrid systems, such as ANFIS [19] and artificial neural network with genetic algorithm (ANN-GA) [20], have also been examined.

Previous research demonstrates a considerable interest in the development of intelligent systems for automatic classification of faults in PV modules. Table 1 provides a general review of the application of several AI approaches in fault detection and diagnosis in PV plants. The previous work is categorized, based on the input datasets, into thermography techniques (Table 1a), PV modules electrical I-V characteristics (Table 1b), PV modules electrical I-V characteristics and environmental conditions (Table 1c), and using thermography and I-V measurements (Table 1d). The input parameters, fault types, classification accuracy, and limitations are summarized for each technique. The previously described methods, however, are restricted to specific fault types and/or a single fault per module. In some cases, the proposed techniques are capable of defining only a faulty module without being capable of detecting the fault type. Additionally, I-V measurements are often carried out when the PV module is offline; this introduces a significant difficulty for automation of fault detection and classification processes. The use of IRT offers the advantage of possible online operation of the diagnosis process. However, to the authors’ knowledge, there is no research work on correlating the infrared (IR) images of faulty modules with their I-V measurements. Such a correlation is very useful in developing an online-detection classification system that is useful for maintenance planning. The importance of accurately classifying faults lies in determining the required maintenance action and calculating the remaining useful life (RUL) of the PV module. Moreover, previous studies in classifying faults have focused on polycrystalline and monocrystalline PV technologies, and barely any of this research has dealt with PV modules of thin-film technology.

To this end, this paper proposes using an ANFIS for automation of fault classification of copper indium gallium selenide (CIGS) thin-film PV modules. The proposed ANFIS approach is based on statistical and mathematical features of outdoor IRT and I-V measurements of PV modules. A correlation matrix is proposed to be developed between the operating power ratio (P_r_) and the effective features extracted from thermal images. The main scientific contributions made in this work are summarized as follows: (1) an automatic ANFIS fault classification technique is developed for the detection and classification of PV module faults; (2) new effective features for each type of fault are identified that are highly correlated with the P_r_ of a PV module; (3) a correlation matrix is developed to address correlations between the P_r_ and the proposed effective features extracted from thermal images. Thus, the need for offline I-V measurements is eliminated, and (4) the effectiveness of the proposed scheme is evaluated using collected datasets from a typical plant with PV modules with multiple faults.

## 2. Methodology

**Figure 1:**
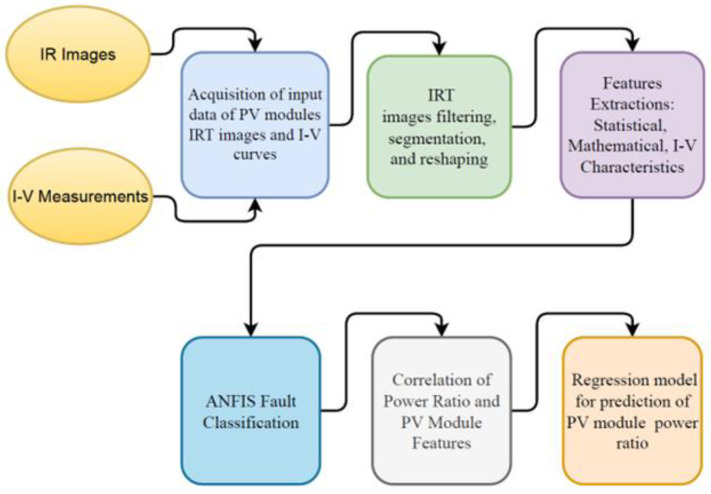
Procedure of development of ANFIS scheme for automation of fault detection and classification.

The procedure implemented in developing the present ANFIS scheme for fault classification using IRT and I-V measurements is shown in Figure 1. The datasets are prepared by acquisition of input data of PV module thermal images and the corresponding I-V curves. Thermal images are processed using image filtering, segmentation, and reshaping. Image filtering and panels reshaping are performed using pixel-shifting techniques. In our methodology, a moving average filter and multidimensional filter are used. The geometric transformations are used to correct distortions caused by viewing geometry using pixel-shifting techniques, and reshape that panel into a rectangular shape. The destination image is filled by regular scan lines, taking the values from the source image by bi-cubic interpolation. This is achieved by applying geometric transformations to images. After getting the rectangular shape, the cropping process is easily completed to remove unwanted segments. The next step uses feature extraction methods. Statistical and mathematical feature extraction techniques of IR images and I-V curves are employed. Once feature extraction techniques have been deployed, ANFIS is utilized to classify CIGS module failures. ANFIS is learnt using the training dataset, just like artificial neural networks. Faults are classified according to their types (A, B, C, D, E, F, and G). The most effective features, correlated with the power ratio, are identified to develop a correlation matrix between these effective features and PV module operating power ratio. A regression model is applied, utilizing the correlation matrix, to predict the PV module’s operating power ratio for each type of fault. The value of the operating power ratio of each module is then used for evaluating the overall plant power and for management of the power plant’s operation and maintenance.

## 3. Collection of Input Datasets

The datasets for this investigation were gathered from a PV power plant located in Bani Mazar, Egypt. The plant consists of 84 TianWei solar films comprised of TW-SF-W100 CIGS thin-film modules. I-V curves were obtained using a variable resistor and a potentiometer. A total of 84 IRT images were captured with a Fluke Ti3 IR camera. The IEC62446 [39] requirements for PV thermography testing were followed. Before the tests, the junction boxes and all electrical connections were inspected. The solar irradiance (E) was around 900 W/m^2^ during the testing, and the ambient temperature was 19 °C, with a low wind speed of about 18 km/hr. Cloud shading was avoided during filming to avoid the appearance of warmer reflected areas and the resulting effect of fault misunderstanding [39]. The emissivity of PV panels was also considered, and all shots were taken from a 50 to 60° angle. The plant’s defective modules were previously identified; most of the modules that were tested contained multiple faults.

**Figure 2:**
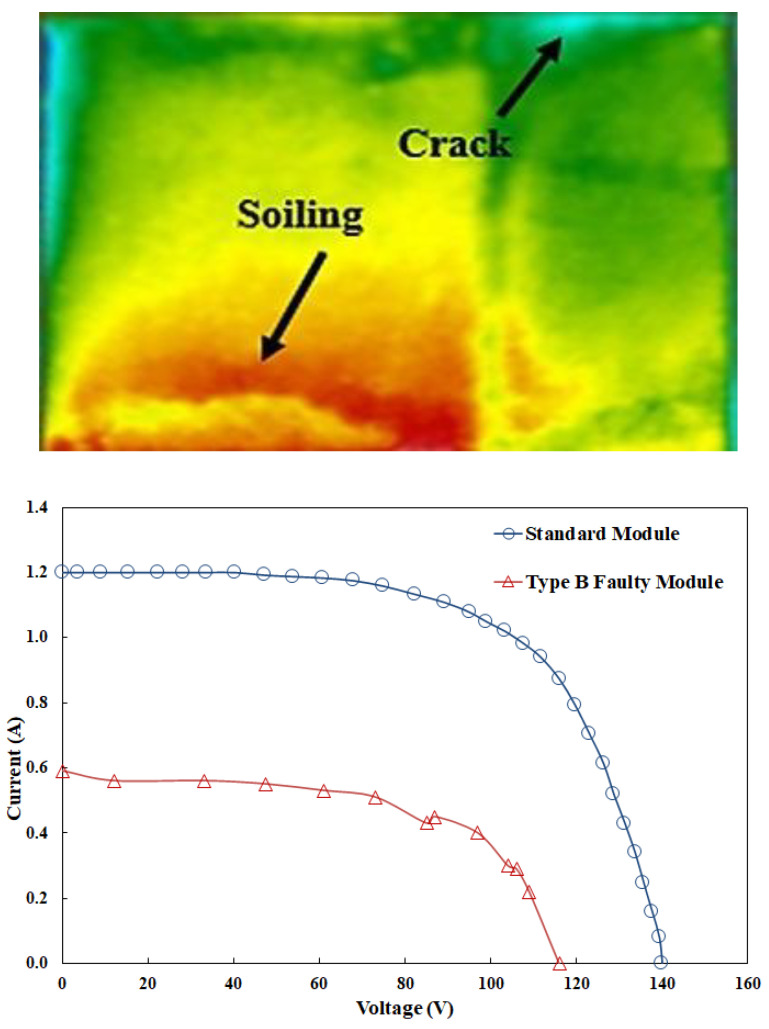
IRT image and I-V curve for a PV module with Type-B fault.

The current study focuses on the most frequent defects that can occur in PV modules. Table 2 shows how the faults are grouped. For each type of fault, an IRT image and I-V measurements were acquired for each of the 84 modules. IRT imaging and I-V measurements were conducted three times for each module to reduce measurement uncertainty. In [3,38], the present authors have published a detailed set of IR pictures and I-V curves. Figure 2 shows an example of IRT image and I-V curve for a PV module with Type-B fault.

## 4. Features Extraction Techniques

The feature extraction techniques applied in this study include statistical feature extraction, mathematical parameters-based feature extraction, and electrical measurements-based feature extraction. The parameters of IR image features are summarized in the following subsections (Section 4.1, Section 4.2 and Section 4.3). The process of features extraction was carried out using the MATLAB platform. Detailed description of these features is reported in [3,38]. The ratio of operating voltage, current, power, fill factor, and efficiency are all features of the I-V characteristic curve. They are derived by comparing the PV module operational value to its healthy equivalent. The investigated module is denoted by the subscript o, while the healthy module is denoted by the subscript h. Because the healthy values of these parameters alter with solar irradiation and electrical demand, they might be considered the maximum operational voltage, current, and power of all PV modules in use. The mean, standard deviation, skewness, and kurtosis are all statistical properties of an IRT image. The capability of employing I-V measurements and statistical aspects of IR images for CIGS module fault classification is discussed in [3]. Statistical features and I-V measurements are used to generate a generic classification matrix for fault identification and diagnosis. Statistical features, on the other hand, have demonstrated a limited ability to distinguish and detect problems in defective modules with many types of defects occurring at the same time. Faulty modules of the types (A, B), (B, E), (D, E), (E, F), (E, G), (E, H), (E, I), and (G, H) are examples.

### 4.1. Statistical Parameters of IR images

The following equations summarize the statistical parameters of IR images features.

(1) $$\bar{\chi }=\frac{1}{N}\cdot \overset{N}{\underset{i=1}{\sum }}\chi_{i}$$

(2) $$\sigma =\sqrt[{}]{\frac{1}{N-1}\overset{N}{\underset{i=1}{\sum }}(\chi_{i}}-\bar{\chi }{)}^{2}$$

(3) $${\;\gamma }_{1}=\frac{1}{N}\overset{N}{\underset{i=1}{\sum }}{\left[\frac{\chi_{i}-\bar{\chi }}{\sigma }\right]}^{3}$$

(4) $$\gamma_{2}=\frac{1}{N}\overset{N}{\underset{i=1}{\sum }}{\left[\frac{\chi_{i}-\bar{\chi }}{\sigma }\right]}^{4}-3$$

where Equations (1)–(4) represent the mean, the standard deviation, the skewness, and the kurtosis of the images’ feature parameters. N is the total number of pixels, $\chi_{i}$ is the pixel value, and σ is standard deviation of the image.

### 4.2. Mathematical Parameters of IR Images

The mathematical parameters of IR include the peak-to-peak value (PP), flatness density measure (FDM), flatness continuity measure (FCM), global form factor (GFF), maximum form factor in vertical and horizontal (FF_maxv_, FF_maxh_), mean form factor (FF_mnv_, FF_mnh_), first-order zero temperature change rate (ω), second-order zero temperature change rate (ώ), and cold area percentages measure (CPM). They are calculated using equations 5 to 17, where N_w_ is the number of rows, N_l_ is the number of columns, T_i,j_ is the pixel temperature at (i, j), and (dTH, d2TH, dTV, d2TV) are the first- and second-order derivatives in the horizontal and vertical axes using ε ≈ 0.01.

(5) $$\mathrm{PP}=T_{max}\;-\;T_{min}$$

(6) FDM=((∑peak pixels)/(totlal number of pixels))

(7) $$\mathrm{FCM}=\frac{\sum \mathrm{peak}\;\mathrm{pixels}}{\mathrm{No}\;\mathrm{of}\;\mathrm{flat}\;\mathrm{Portions}}$$

(8) $$\mathrm{GFF}=\frac{N\times \mathrm{peakvalue}}{\sum_{j=i}^{\mathrm{Nl}}\sum_{i=1}^{\mathrm{Nw}}T_{i,j}}$$

(9) $$FF_{maxv}=\frac{N_{l}\times \max\left\{\max_{j}T_{i,j}\right\}}{\sum_{i=1}^{N_{L}}\left\{\max_{j}T_{i,j}\right\}}$$

(10) $$FF_{maxh}=\frac{N_{w}\times max\left\{max_{i}\left\{T_{i,j}\right\}\right\}}{\sum_{j=1}^{N_{w}}\left\{max_{i}T_{i,j}\right\}}\;\;$$

(11) $${\mathrm{FF}}_{\mathrm{mnv}}=\frac{N_{l}\times \max\left\{\sum_{i=1}^{N_{w}}T_{i,j}\right\}}{N_{w}\times \sum_{j=1}^{N_{i}}\sum_{i=1}^{N_{w}}T_{i,j}}$$

(12) $${\;F}_{\mathrm{mnh}}=\frac{N_{w}\times \max\left\{\sum_{j=1}^{N_{l}}T_{i,j}\right\}}{N_{l}\times \sum_{i=1}^{N_{w}}\sum_{j=1}^{N_{l}}T_{i,j}}$$

(13) $$\omega_{v}=\frac{\mathrm{No}.\mathrm{of}\{\mathrm{dTV}<\varepsilon \}}{N},\;\omega_{h}=\frac{\mathrm{No}.\mathrm{of}\{\mathrm{dTH}<\varepsilon \}}{N}$$

(14) $${\bar{\omega }}_{h}=\frac{\mathrm{No}.\mathrm{of}\left\{d2\mathrm{TH}<\varepsilon \right\}}{N},\;{\bar{\omega }}_{V}=\frac{\mathrm{No}.\mathrm{of}\left\{d2\mathrm{TV}<\varepsilon \right\}}{N}$$

(15) CPM=((∑coldpixels)/(totlal number of pixels))

### 4.3. Electrical Parameters of I-V Measurements

The electrical parameters are calculated using Equations (16)–(20). They include the operating current ratio (I_r_), operating voltage ratio (V_r_), operating power ratio (P_r_), fill factor (FF), and PV efficiency (η). P_in_ is the incident solar radiation and A is the module area. Subscripts o and h refer to the operating value and the value of the healthy PV module.

(16) $$I_{r}=I_{o}/I_{h}$$

(17) $$V_{r}=V_{o}/V_{h}$$

(18) $$P_{r}=P_{o}/P_{h}$$

(19) $$\mathrm{FF}=(I_{o}\;\times \;V_{o})/(I_{\mathrm{SC}}\;\times \;V_{\mathrm{OC}})$$

(20) $$H=(I_{\mathrm{SC}}\;\times \;V_{\mathrm{OC}}\;\times \;\mathrm{FF})/(P_{\mathrm{in}}\;\times \;A)$$

The use of mathematical parameters for IR image features extraction was studied in [38]. These features are calculated using the pixel values of an IR image, as detailed in Section 4.1. The physical significance of each of these features is explained in [38]. The GFF, which analyses the temperature variation of pixels depending on the type of fault, is found to be one of the most useful parameters of the diagnosis technique. The temperature of modules varies depending on the fault type: low for dirty modules, moderate for PID fault modules, and high for hot spot modules. For dirty modules, the average value of GFF rises when multiple faults are observed. Assessing the maximum and mean temperature variation in both the horizontal and vertical axes improves the accuracy of fault diagnosis.

The proposed features are demonstrated to be independent of temperature fluctuations that may arise owing to the use of different infrared camera sensors because they do not directly deal with pixel temperatures or values. They can also describe the shape of fault patterns in cold and hot areas. A broad classification matrix summarizes the relationships between fault type and associated variables. This matrix can be used to schedule maintenance and automate fault classification.

## 5. ANFIS Fault Classification Technique

After deploying feature extraction methods, ANFIS is used to categorize CIGS module faults. Fuzzy logic and ANNs are combined in ANFIS. Fuzzy if-then rules with appropriate membership functions are derived using the ANN’s learning capabilities [40]. The ANFIS structure and its implementation of automatic fault detection are described in the following subsections.

### 5.1. ANFIS Structure

**Figure 3:**
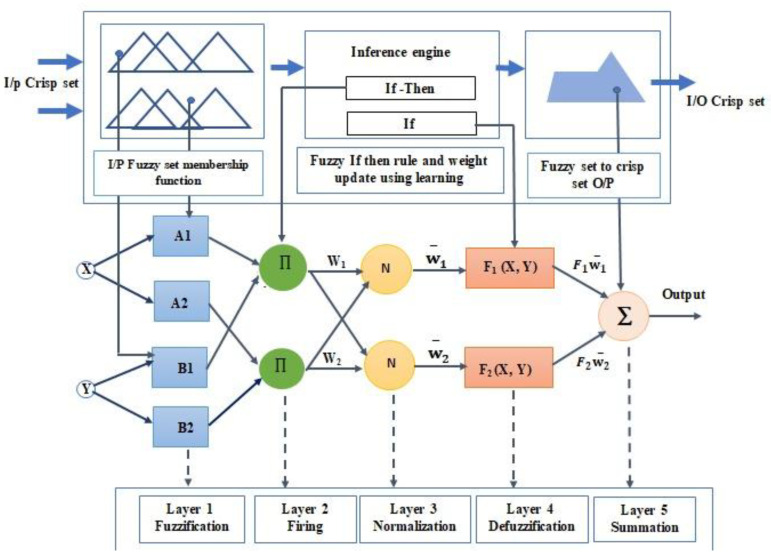
Proposed ANFIS structure.

A typical ANFIS structure with two input variables and five levels is shown in Figure 3. From a set of inputs, the ANFIS algorithm produces only one output. The output is determined through consecutive stages including establishing fuzzy rules, fuzzifying the inputs with membership functions, defining rule strength and assessing its implications with a specific input dataset. The curve’s parameters of the membership functions are determined using product values among the preset learning rules and the relevant weighted values. The parameters of membership functions are used to calculate the ratio between the individual and total weighted values. Finally, ANFIS foresees the target by calculating an overall gain value, which serves as the output value.

To attain the desired values, the input and output membership function parameters are adjusted during the learning process. The ANFIS method is a hybrid algorithm that employs both back-propagation and least-square estimation techniques. These techniques are combined in an artificial neural network to offer a considerably faster and more accurate output to the ANFIS target. The linguistic values of the relationship between the input data rows (variable data) are determined by the rule “IF-THEN”.

The ANFIS design architecture comprises two inputs (x, y), two rules (R1, R2), and five layered feed-forward networks, as shown in Figure 3. The design also indicates adaptive (square) and non-adaptive (circle) nodes with one output. The learning algorithm is used to update the adaptive node parameters by minimizing tracking errors between target data and ANFIS output. The two fuzzy if-then rules are determined as function of the inputs (x, y) [40]. A1, B1, A2, and B2 are the fuzzy sets and F is the number of membership functions. A typical ANFIS structure includes five layers. Layer 1 is the fuzzification layer, and every node I is an adaptive node in this layer. This layer’s outputs are the inputs’ fuzzy membership grades. Layer 2 is called the rule layer (the firing). It is a fixed node layer, labelled ∏. To obtain the output of layer 2, which is the product of all the incoming signals, the firing strength is computed. Layer 3 is the normalization layer. It has a fixed node, labeled N. Normalized firing strengths $w_{i}$ are the outputs of this layer. It determines the ratio of the i^th^ rule’s firing strength to the total firing strength. Layer 4 is the defuzzification layer; it is a part that takes the fuzzy set inputs generated by the inference engine and transfers the fuzzy values into crisp values. Layer 5 is a summation neuron, a stationary node that adds all input signals together to compute the final output.

### 5.2. Application of ANFIS for Classification of Faults

The Jang model [40] is adopted in the present study; it is one of the most common fuzzy system architectures with less time-consumption and works well when large data are available for training. The input data are built from the feature extraction methods described in Section 4. The available input data are randomly divided into 50% as training data, 25% as check data, and 25% as test data. At the beginning, input and output datasets are used to plan the ANFIS architecture. Using feature extraction methods, the principal component analysis (PCA) is estimated for each input dataset. PCA is a technique for reducing the dimensionality of a dataset containing many interconnected elements. This was achieved by using a covariance matrix of extracted features to extract the more effective features that are uncorrelated with one another. Therefore, features reduction using principal component analysis decreases the computation volume and enhances the classification accuracy. According to the present results, the form factors (GFF, FF_maxh_, FF_maxv_, FF_mnh_, FF_mnv_, flatness continuity measure (FCM), statistical features extraction, operating power ratio (Pr) and efficiency (η) are found to be the most effective features.

The data extracted using principal components analysis are divided into three categories for each feature extraction method: training, checking, and testing. The goal of utilizing a checking dataset is to avoid overfitting the training dataset. In theory, as training advances, the model error tends to decrease, leading to overfitting, and an abrupt increase in model error occurs. Overfitting is accounted for by testing the FIS trained on the training data against the checking data. Furthermore, if these errors indicate model overfitting, the membership function parameters associated with the smallest checking error are chosen. The testing dataset is used to evaluate the FIS’s generalization capability. Eight membership functions are selected for inputs and the same type of membership functions for the output is applied during the generation of a fuzzy inference system. The objective of increasing the number of fuzzy memberships per variable is to improve the matching accuracy of the proposed ANFIS. As the number of fuzzy memberships per variable increases, the number of robust rules increases, resulting in higher matching accuracy. The total number of samples (PV modules) is slightly low, 84 samples, compared to other ANFIS applications; thus, the number of fuzzy memberships is increased to overcome this problem. The system was prepared with training data and checked with a test dataset. The ANFIS model is implemented using MATLAB^®^ software. The number of nodes and fuzzy rules is determined, and a neural network training algorithm is utilized to refine the rules and decrease errors. Finally, the output of the classifications is obtained.

### 5.3. Analysis of ANFIS Results

In this section, the results of the performance of ANFIS models, to classify the faults of CIGS PV module, are illustrated. For each type of feature extraction, a series of trials was applied to the dataset to select an adequate membership function type for the best classification. The data were divided into three sets, each of which was mutually exclusive. There were 512 rules, three inputs, one output, and eight membership functions per input in the final network. The ANFIS system parameters are shown in Table 3. The results of each feature extraction method are addressed in the following section. After many trials, the best membership function type that obtained a high classification percentage for each type of feature extraction is shown in Table 4. Also, all features were trained and tested together.

A general classification matrix (GCM) was proposed in [3,38] for classification of faults. It has been shown that mathematical parameter-based feature extraction exhibited a higher capability in classification than statistical feature extraction and electrical measurement-based feature extraction. However, the GCM takes more time in classification, since it compares each type of fault with other types using feature extraction methods. On the other hand, the ANFIS approach proposed in the present study is very fast in the classification process and detects the type of fault for the classified module. For each type of fault, the proper required maintenance action can be scheduled/implemented once the data are trained. This represents an advantage as compared to previous studies on fault classification that define only the module to be either defective or non-defective and fails to determine the fault type and corresponding proper maintenance action.

## 6. Estimation of Operating Power Ratio

**Figure 4:**
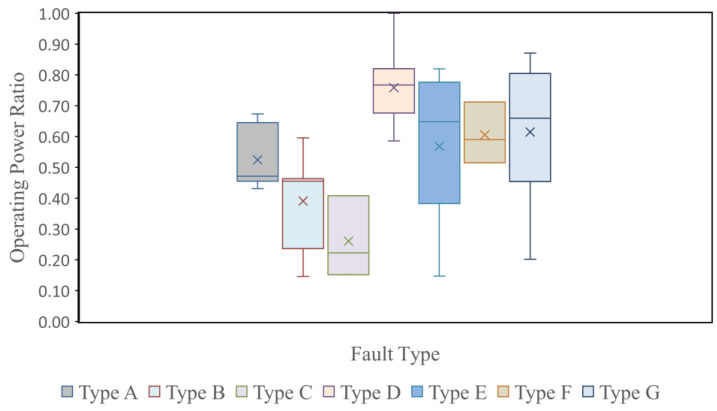
Box plot of operating power ratio for different fault types.

The operating power ratio (P_r_) of PV modules represents the ratio of PV module power output and the healthy module power output (refer to Equation (18)). The previously reported research efforts were concerned with the utilization of IRT images for the detection and classification of PV faults. No research efforts have considered the possibility of correlating the features of IRT images to the operating power ratio. Such a correlation is very useful in predictive maintenance applications; it can be used as a measure for deciding a maintenance action or even module replacement. Assessment of the operating power ratio of a given PV module in the plant is normally performed using I-V measurements in offline conditions. Correlation of IRT image features to the P_r_ of PV modules can be performed online without any disturbance in plant operation. Studying the effect of faults on power loss is very useful to correlate fault types with module reliability, degradation, and remaining useful life, and obtaining a future picture of the performance of the PV power plant. Figure 4 shows a box plot of power loss due to faults, with the *Y*-axis representing the operating power ratio of CIGS PV modules and the *X*-axis representing the box plot of each type of fault.

**Figure 5:**
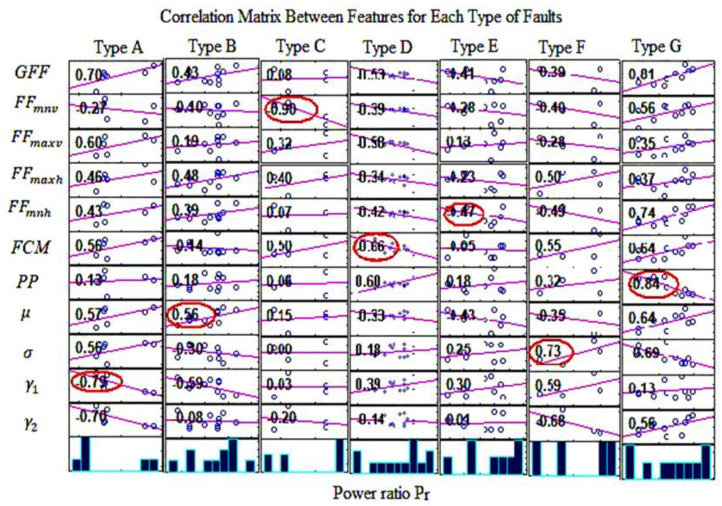
Correlation matrix between features and operating power ratio for each type of fault.

In the present article, the correlation between the P_r_ and the mathematical and statistical features is obtained for each type of fault. The features with high correlation with the power ratio are determined. Then, regression analysis is used to obtain the correlation equation between the high correlation features and the operating power ratio using Minitab 19 software. Figure 5 shows the correlation matrix between features with the P_r_ for each type of fault. The high correlation values are marked with red circles. For each type of fault, there is a feature that gives a high correlation with the P_r_. Since the maintenance action of faulty modules with fault type H is to remove the module, this type of fault was not considered in the correlation with the P_r_. It can be observed that the features that have a high correlation with the P_r_ include skewness ($\gamma_{1}$), FDM, ω, and PP.

**Figure 6:**
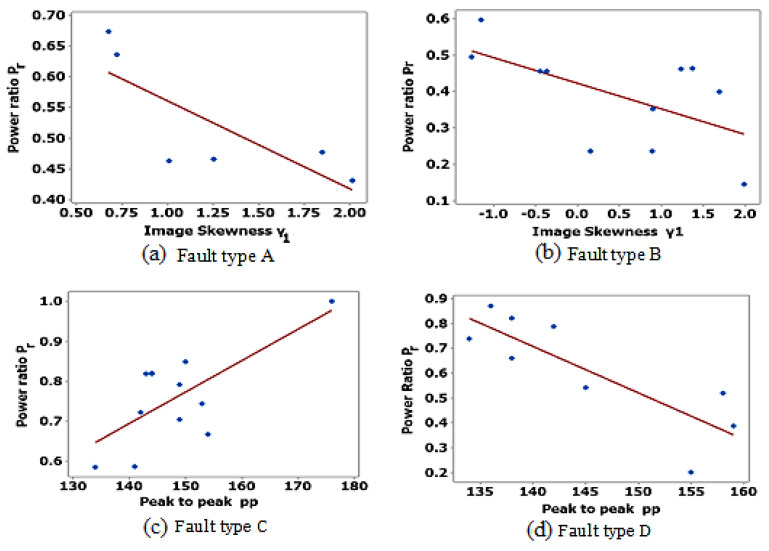
Regression analysis for correlation of operating power ratio for different types of faults.

The results of regression analysis using Minitab software are shown in Figure 6. The regression model equation and the values of the R-squared and *p*-value for each fault type are shown in Table 5. The value of R-squared (0–100%) represents the scatter around the regression line. A *p*-value between 0 and 1 is commonly used to represent the level of statistical significance. The lower the *p*-value, the more evidence there is for rejecting the null hypothesis. A statistically significant *p*-value is usually less than 0.05. the combination of low *p*-value and high R-squared indicates that changes in the predictors are related to changes in the response variable and that the regression model explains a lot of the response variability. The combination of low *p*-value and low R-squared indicates that the regression model has significant variables but explains little of the variability. The results in Table 5 reveal that features with faults of type A, C, D, F, and G have high R-squared values. Type B and F faults, on the other hand, have low R-squared values. All types of faults have a low *p*-value, indicating strong evidence against the null hypothesis. The current study advises that more research should be carried out on the analysis of the operational power ratio for type B and F faults. Figure 6 shows that the values of P_r_ decrease with the increase in $\gamma_{1}$ for A and B type faults. The variation of P_r_ with PP is dependent upon the fault type. The value of P_r_ decreases with the increase in PP for fault type D and decreases with the decrease in PP for fault type C.

## 7. Conclusions

An ANFIS is developed for automation of the fault classification process of PV modules. Statistical and mathematical features of outdoor IRT and I-V measurements of thin-film PV modules of a typical CIGS PV plant are used to provide the dataset for the implementation and analysis of the proposed scheme. The proposed features are proven to be unaffected by temperature variations that may occur due to the usage of different infrared camera sensors.

Many tests have been carried out to determine the best type of membership function for high classification accuracy. PCA is used to reduce dimensionality and speed up the classification process. This was achieved by using a covariance matrix of extracted features to obtain the more effective features that are uncorrelated with one another. According to the results, the most effective classification features are found to be global form factor (GFF), maximum form factor for horizontal and vertical (FF_maxh_, FF_maxv_), mean form factor for horizontal and vertical (FF_mnh_, FF_mnv_), flatness continuity measure (FCM), statistical features, operating power ratio (P_r_) and efficiency (η). The evaluation of the proposed approach shows that the accuracy of classification reaches 100% using feature extraction methods that are based on mathematical parameters and I-V measurements, and 83.33% using features based on statistical parameters.

A novel approach is proposed for developing a correlation matrix between the P_r_ and the effective features extracted online from IRT images. Thus, the need for offline I-V measurements to estimate the P_r_ of PV modules is eliminated. Effective features that are highly correlated with the operating power ratio of PV modules are determined for each type of fault. The features of IR images that have a high correlation with the P_r_ include skewness ($\gamma_{1}$), mean (μ), mean form factor in the vertical direction (FF_mnv), FCM, mean form factor in the horizontal direction (FF_mnh), σ, and PP. The results of a regression analysis of the P_r_ demonstrate that features with faults of type A, C, D, F, and G have high R-squared values. Type B and F faults, on the other hand, have low R-squared values. All types of faults have a low *p*-value, indicating strong evidence against the null hypothesis. The present analysis suggests the need for further research work on the analysis of P_r_ for faults of types B and F.

## Figures and Tables

**Table 1:** An overall review of using AI techniques for fault detection and diagnosis in PV plants.

Methodology	Reference	Fault Classification	Accuracy of Classification	Remarks
**(a) using thermography techniques.**				
Texture feature extraction (TFE) and support vector machine (SVM)	[12]	Cracks, hot spots due to shading and soiling. Categorize solar modules into defective and non-defective.	97%	- Limited to a single type of fault per PV module. - Only categorize modules into defective and non-defective - No correlation with module output power
K-nearest neighbor (KNN	[21]	Categorize solar modules into defective and non-defective.	80.3%	
Support vector machine (SVM)			56.8%	
Neural network			92.8	
Support vector machine (SVM)	[22]		91.2%	
Deep-learning convolutional neural network (CNN)	[22]		89.5%	
n Bayes: a binary class density-based classifier	[23]		98.4%	
The automated edge detection technique	[24,25]	Defective solder junctions, short circuits, and bypassed substrings.	Not reported	
Deep learning neural network	[26]	Cracks, shadowing, diode, soiling, hotspots, and offline module.	Classify 12 anomaly types with an average of 86%	
**(b) with input datasets from PV modules electrical I-V characteristics.**				
Multi-class adaptive neuro-fuzzy classifier	[19]	Partial shading, increased series resistance, bypass diode short-circuited, bypass diode impedance, PV module short-circuited.	65–100% depending on fault type	- I-V datasets obtained using a real time PV emulator. Datasets from real systems are not considered.
Principal component analysis (PCA)	[27]	Shading faults.	97%	- Real systems configurations are not considered.
AI nonlinear autoregressive exogenous neural network (NARX)	[28]	Open and short-circuit degradation, faulty MPPT, partial shading (PS).	98.2%	- Applicable for small systems.
Multilayer neural network with a scaled conjugate gradient algorithm (SCG)	[29]	Short circuits, aging, shading faults, and bypass diode faults.	99.6%	- Consider polycrystalline and thin-film PV technologies.
Convolutional neural networks (CNN)	[30]	Partial Shading (PS), high impedance, low location mismatch, maximum power point tracking (MPPT).	73.53%	- 2D scalograms generated from I-V characteristics using continuous wavelet transform. (CWT) - Low classification accuracy.
Multiclass adaptive boosting (AdaBoost) algorithm, using multiclass exponential (SAMME) loss function based on the classification and regression tree (CART)	[31]	Short-circuit faults (SCF), partial shading with the bypass-diode on (PSBO), partial shading with the bypass-diode reversed (PSBR), and abnormal aging faults (AAF).	99.4%	- The results consider only faulty modules. - Time-consuming, suitable for a small number of modules.
Radial basis function (RBF) kernel extreme learning machine (ELM) optimized by simulated annealing algorithm,	[32]	Short circuits, shading faults, and aging.	Shadows 91.55%	Need real outdoor experiments.
			Short circuits 93.64%	
			Aging 90.91%	
Artificial neural network	[33]	Partial shading	Not reported	A single type of fault.
Multiclass adaptive neuro-fuzzy classifier (MC-NFC) and ANN	[19]	Partial shading, high series resistance, bypass diode impedance and short circuits.	Not reported	The MC-NFC outperforms the ANN-classifier.
**(c) with input datasets from PV modules electrical I-V characteristics and environmental conditions.**				
Backward propagation NN optimized by genetic algorithm	[20]	Short circuits, local material aging, shading.	78% for short circuits, 97% for aging, 100% shadows	- Expensive real time PV modules output current, voltage, irradiation, ambient temperature monitoring system for each module. - Limited number of faults.
Neuro-fuzzy and simulation	[34]	Upper and lower earth faults, diode short-circuit faults, partial shading.	Not reported	Limited number of PV module circuit faults.
Cursive linear model and an ANN	[35]	Short circuits, open circuits, partial shading, and degradation.	92.64%	Limited number of PV module circuit faults.
ANNs	[36]	Disconnected modules.	97%	- Using solar radiation and PV measured output power. - Limited to one type of fault.
**(d) with input datasets from thermography analysis and PV modules electrical I-V characteristics.**				
Statistical features extraction and electrical measurements characteristics	[3]	Cracks, delamination, burn marks, PID, soiling, and open strings.	Not reported	Applied for CIGS PV modules.
Fuzzy inference system (FIS) using Mamdani-type fuzzy controller	[37]	Identify the six main types of hotspots that influence PV modules.	96.7%	Inability to detect hot spots when there is a lot of partial shading.
Novel feature extraction based on mathematical parameters	[38]	Cracks, delamination, burn marks, PID, soiling, and open strings.	Not reported	Detect all types of CIGS thin-film PV modules, detect modules with multi-faults.

**Table 2:** Categories of CIGS PV module faults.

Category/Type	Description
**A**	Soiling
**B**	Cracking and soiling
**C**	Cracks, burn marks, and soiling
**D**	Potential-induced degradation (PID)
**E**	PID and cracks
**F**	PID, cracks, and delamination
**G**	Open strings (HM)
**H**	Dead modules

**Table 3:** ANFIS system parameters.

Item	Number of
Nodes	1078
Linear parameters	2048
Nonlinear parameters	48
Training data pairs	36
Checking data pairs	27
Fuzzy rules	512

**Table 4:** Type of membership and accuracy for type of feature extraction method.

Type of Feature Extraction (FE) Methods	Type of Membership Function	Accuracy
Statistical (FE)	Triangle	83.33%
I-V measurement (FE)	Gaussian	100%
Mathematical parameter (FE)	All type	100%

**Table 5:** Regression model parameters for different types of faults.

Fault Type	Regression Model	R-sq %	*p*-Value
A	$P_{r}=0.7035-0.1428\;\gamma_{1}$	62.34	0.062
B	$P_{r}=0.4227-0.07026\;\gamma_{1}$	34.83	0.056
C	$P_{r}=0.5929-19.28\;\mathrm{FDM}$	99.99	0.06
D	$P_{r}=-0.4062+0.007860\;\mathrm{pp}$	48.67	0.012
E	$P_{r}=-5.292+6.747\;\omega$	57.41	0.029
F	$P_{r}=0.5444+0.6615\;\mathrm{FDM}$	77.43	0.315
G	$P_{r}=3.332-0.0187\;\mathrm{pp}$	69.24	0.005

## Data Availability

The data that support the findings of this study are available from the corresponding author, upon reasonable request.
